# Evaluation of asbestos dispersion during laser ablation of rocks containing Naturally Occurring Asbestos (NOA)

**DOI:** 10.1016/j.heliyon.2024.e39624

**Published:** 2024-10-22

**Authors:** Andrea Bloise, Francesco Parisi, Mauro Francesco La Russa, Carmine Apollaro, Nicolas Godbert, Iolinda Aiello, Eugenia Giorno, Alessandro Croce, Laura Cagna, Ana Jesús López, Alberto Ramil, Dolores Pereira

**Affiliations:** a Department of Biology, Ecology and Earth Sciences, University of Calabria, 87036, Rende, CS, Italy; b University Museum System – SiMU, Mineralogy and Petrography Section, University of Calabria, 87036, Rende, CS, Italy; c MAT_InLAB (Laboratory of Inorganic Molecular Materials), Center of Excellence CEMIF.CAL, LASCAMM CR-INSTM of Calabria, Department of Chemistry and Chemical Technologies, University of Calabria, 87036, Rende, CS, Italy; d LPM-Materials Preparation Laboratory, STAR-Lab, University of Calabria, Via Tito Flavio, 87036 Rende, CS, Italy; e Department of Science and Technological Innovation, University of Eastern Piedmont, Viale Teresa Michel 11, 15121, Alessandria, Italy; f Research Laboratories, Research, Training and Innovation Infrastructure, Department of Integrated Research and Innovation Activities (DAIRI), SS. Antonio e Biagio e Cesare Arrigo University Hospital, Via Venezia 16, 15121, Alessandria, Italy; g Ferrol Industrial Campus, University of A Coruña, 15471, Ferrol, Spain; h Department of Geology, University of Salamanca, 37008, Salamanca, Spain

**Keywords:** Asbestos, Tremolite asbestos, Actinolite asbestos, Chrysotile, HEPA filters

## Abstract

Health risks are often overlooked when the short-term consequences are not immediately apparent. During restoration work, cleaning actions can generate particles that pose health risks to workers through inhalation. This is particularly true in the case of asbestos fibres that might be spread out from the laser cleaning of buildings or heritage artifacts made of stone, such as serpentinite and other ultramafic rocks, that have a high probability of containing asbestos (e.g., chrysotile, tremolite asbestos, actinolite asbestos). To show workers the importance of wearing proper protection to prevent health injuries, several serpentinite samples, ascertained to contain asbestos minerals by specific investigations, have been laser ablated using ad hoc modified equipment in order to collocate a HEPA filter prone to collect all dust emitted during ablation. The powder deposited on the surface of these filters after laser ablation was analyzed, by Powder X -ray Diffraction (PXRD), Transmission Electron Microscopy combined with Energy Dispersive Spectroscopy (TEM/EDS), micro-Raman spectroscopy (μ-Raman) and Fourier-transform infrared spectroscopy (FTIR/ATR). The results confirmed the presence of asbestos fibres during the laser ablation of rocks containing Naturally Occurring Asbestos (NOA), emphasizing the importance of wearing appropriate personal protective equipment (PPE) during these procedures. Noteworthy, approximately 33 % of the analyzed fibres met the WHO criteria in size for respirable fibres. Furthermore, through our experiments, we also demonstrated that using tools that integrate filters into working tools would definitively further decrease the risk of fibres inhalation to workers.

## Highlights

• Asbestos released during laser ablation processes poses a significant hazard.
• HEPA filters and hygienic face masks effectively capture asbestos.
• Filtration methods can mitigate the risk of asbestos inhalation.
• μ-Raman and FT-IR are highly reliable techniques for identifying asbestos in HEPA filters.
• Disseminating these findings to workers will promote self-protection measures.

## Graphical abstract

**Figure:**
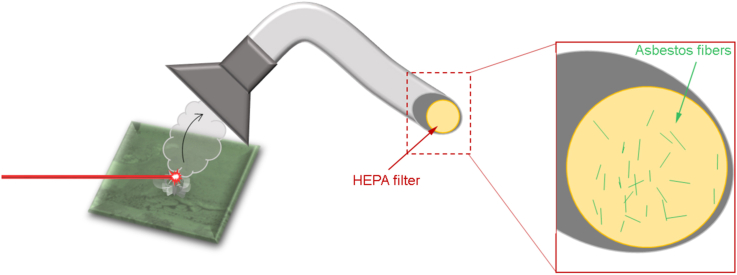

## 1 Introduction

The term "asbestos" refers to a group of minerals that includes one fibrous serpentine mineral (chrysotile) and five fibrous amphiboles (amosite, anthophyllite asbestos, actinolite asbestos, tremolite asbestos and crocidolite). Asbestos exposure can be associated with several different lung injuries, including asbestosis, bronchitis, respiratory diseases, lung cancer and mesotheliomas. It was not until the late 1950s that asbestos was recognized as a human carcinogen. This danger is associated with the fibrous nature of asbestos minerals, particularly in their respirable form, defined as fibres longer than 5 μm, narrower than 3 μm, and with an aspect ratio greater than 3:1 [1,2]. Additional information on specific terminology, including fibre, fibrils, asbestiform, and elongated mineral particles (EMP), is provided in the Supplementary Materials [[1], [2], [3]]. The International Agency for Research on Cancer [4] classified asbestos as Group 1 “substances carcinogenic to humans”. Since the awareness of asbestos toxicity, the mechanism by which asbestos causes cancer has been intensively studied, although a full understanding has still not been reached [5]. Serpentinite, metabasite and ophicalcite are natural rocks in which asbestos can grow in veins [6]. Owe to their exotic, colour and texture, serpentinite, metabasite and ophicalcite are marketed with the trade name of Green Stones or Green Marbles. Indeed, due to their captivating aesthetic qualities, they have been used since ancient times as popular gemstones, architectural materials and ornamental stones [7,8]. However, for the eventual presence of asbestos fibres, the extraction and processing of serpentinite and metabasite are nowadays subject to regulations that vary depending on the country and region, but that leads to strict regulations and restrictions in many places [9]. Indeed, it is well-established that inhaling asbestos fibres can have deadly consequences for human health [[10], [11], [12], [13], [14], [15]]. For a long time, most epidemiological studies have been based on the study of subjects exposed to asbestos for professional reasons as these were considered the main cause of the pathologies detected in them. There are specific potential occupational exposures to asbestos beyond the typical ones, such as the production of asbestos-containing materials (ACM), and remediation work. Moreover, the risk of inhaling fibres is also related to those individuals who are not directly involved in the processing but who may live near the areas where the processing of these materials takes place (e.g., quarries, stone manufactories) and who unknowingly breathe in the potentially harmful airborne particles. Asbestos can be released by varying weather conditions, such as freezing and thawing, temperature fluctuations, as well as rain and snow, all conditions that may contribute to the erosion of rocks containing Naturally Occurring Asbestos (NOA) and promote the release of natural asbestos fibres in the surrounding areas. As a result of all these processes, dust carried by the wind may trigger an increase in fibres in the air, which if breathed, can affect human health [16,17]. In addition, human activities such as walking on unpaved surfaces or driving, mining, excavation, road construction and agricultural activities can degrade NOA rocks which can cause airborne dust generation in the surrounding environment [6,18,19]. It has been well documented that NOA may increase air pollution if particles are disturbed. For example, asbestos-related contamination has been detected in areas where asbestos has been mined in the past, in areas close to urban settlements or related to new road construction, and around manufacture/buildings built on rocks containing NOA [[20], [21], [22]]. Another source of danger to consider is the potential release of asbestos when producing artifacts made of NOA-containing materials (i.e., garden fountains, pyxes, statues, etc.). However, potentially more important and often overlooked is the eventual release of asbestos fibres during the restoration of stone buildings or facades, and art sculptures, using laser ablation. In this regard, our research group has recently carried out research activities to study the release of chrysotile fibres after laser treatment of rocks containing NOA [23]. Indeed, nowadays the most used technique for cleaning artifacts and walls in restoration is based on laser ablation [24,25]; furthermore, lasers are also used to improve the accuracy of rock cutting and drilling in mining operations [26]. In this preliminary research work Pereira et al. [23], analyzed the chrysotile content of a mother rock of serpentinite and showed that after laser treatment, a non-negligible amount of fibres of chrysotile were retained in the HEPA filters. Through the present study, we aim to complete this study and probe the eventual release of asbestos fibres (chrysotile, tremolite asbestos and actinolite asbestos) that may be released from the laser ablation of serpentinite, metabasite and ophicalcite. It is worth remembering that the thermal stability and strength of amphiboles (tremolite asbestos and actinolite asbestos) in general, are higher than those of chrysotile fibres [[27], [28], [29]]. For this reason, we use commercially available serpentine, metabasite and ophicalcite that were sampled in quarries of the Mount Reventino Unit (Calabria Region, Italy) containing chrysotile, tremolite asbestos and actinolite asbestos. Indeed, in the Calabria region, NOA-bearing materials were extracted for several years, excavating blocks that were commercialized for several purposes. Currently, these quarries are closed due to legal restrictions on the extraction and processing of rocks containing asbestos. In Calabria, the mortality data archive of the National Institute of Statistics (ISTAT) and National Mesothelioma Registry (ReNam) present 163 cases of malignant mesothelioma occurring between 2005 and 2015, yielding an average of about 15 cases per annum [30]. However, it should be noted that the region Calabria has an incomplete database [31]. The calabrian serpentinite, metabasite and ophicalcite used in this research contain both chrysotile and amphiboles (tremolite asbestos and actinolite asbestos) and their exact content certifying the presence of NOA was previously reported [32,33]. Environmental contamination in the Calabria region (South Italy) resulting from the inhalation of asbestos fibres was ascertained by examining the biological tissue of animals living within the selected areas [34]. To evaluate the potential environmental spread of rock fibres, Campopiano et al. (2020) [35] analyzed lung samples from 15 sentinel animals (five sheep, six goats and four wild boars) in Mount Reventino (Calabria, southern Italy). High levels of tremolite fibres were found in almost all the samples of lung tissue, with only two samples from sheep and goats showing sparse antigorite fibres and no chrysotile fibres. Analyses performed on the animals' lungs coincide with the main fibrous constituents found in samples collected around Mount Reventino (in the neighboring municipalities of Platania, Martirano Lombardo, San Mango D'Aquino, Conflenti and Gimigliano) belonging to the tremolite-actinolite series followed by fibrous antigorite [6,29,36,37]. Several municipalities have been identified with a notable surplus of lung diseases cases, like the municipality located on Mount Reventino, due to the presence of natural asbestos [36]. Notably, all these municipalities are situated within the identified risk areas for ophiolite outcrops. The herein study is encompassed within the context of environmental asbestos pollution, which is triggered by the disturbance of materials containing NOA. Our focus deal with the possibility of air dispersion of asbestos fibres released during laser processes that can disturb NOA. The purpose of this study is to qualitatively evaluate the release of asbestos when serpentinite, metabasite and ophicalcite are subjected to laser cleaning. This may happen during the restoration of historic buildings, sculptures, fountains or objects built from these materials. It should be noted that a “controlled use” of chrysotile is permitted in 65 % of countries in the world [9] when the stone sector activities require mining chrysotile and manufacturing (e.g., cutting the stone). In these scenarios, the presence of chrysotile is permitted [38,39]. Another aspect to consider is that, so far, no threshold or safe level of asbestos fibre exposure has been established to guarantee that the risk of cancer is negligible below that level. Through this extended study, we want to increase the knowledge by relating the risk to human health with the environmental exposure to natural asbestos outcrops and increasing the level of awareness on those subjects that are still unknown or too often disregarded. Workers at quarries or restoration interventions are exposed to such dangerous material and the results of this specific study could help to increase the risk perception in workers from the stone sector, but also to construction workers that work in an environment full of dust, keeping in mind that inhalation of fibrous minerals can also take place in environments where there is a dispersion of such fibres even if there is no working activity. In this case, we could talk of risk exposition under non-occupational contexts [40]. Once again, a correct risk perception can enhance security levels across all work areas.

## 2 Materials and methods

### 2.1 Samples

**Fig. 1:**
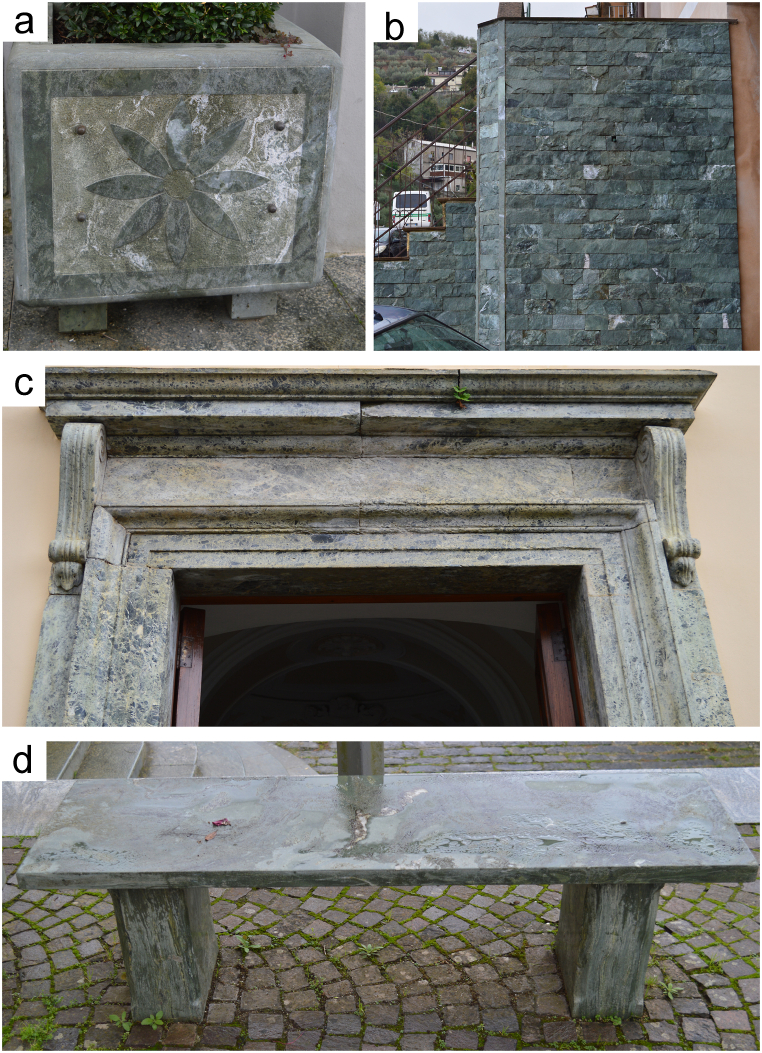
Artifacts with serpentinite and metabasite rocks, (a) planter, (b) wall built with serpentinite blocks, (c) pediment of a metabasite church and (d) metabasite bench.

**Fig. 2:**
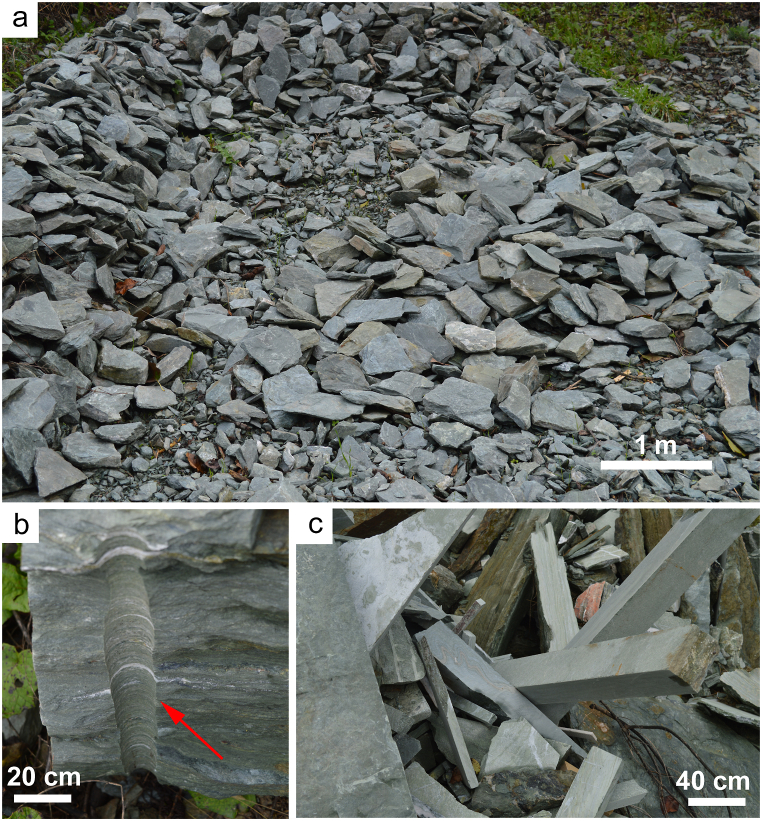
(a) processing waste (tailings fan) of abandoned serpentinite quarry, Conflenti (Italy), (b) drilling details of serpentinite rock (indicated by the red arrow) and (c) serpentinite rocks cut for construction uses.

Five slabs of commercial metabasite, serpentinite and ophicalcite were sampled in quarries of the Mount Reventino Unit (Calabria Region, south Italy). The main exploitation places are located near Gimigliano (South-eastern Sila Piccola, Calabria, Italy), Martirano and in the Mount Reventino area (South-western, Sila Piccola, Calabria, Italy). The samples selected for this study have been the subject of previous research on NOA in the Calabria Region (South Italy), so that they are well characterized regarding mineralogy and geochemistry [6,29,32,41]. Serpentinite, metabasite and ophicalcite rocks are extracted for use as building and ornamental stones, as well as for crafting ornamental artifacts such as stoups, vases, and sculptures (Fig. 1). Our methodology involved applying laser ablation to fully characterized samples. This approach simulates activities such as drilling or cutting these rocks (Fig. 2). We utilized a filter with the laser equipment to observe how various particles are captured during the ablation process.

### 2.2 Laser ablation

**Fig. 3:**
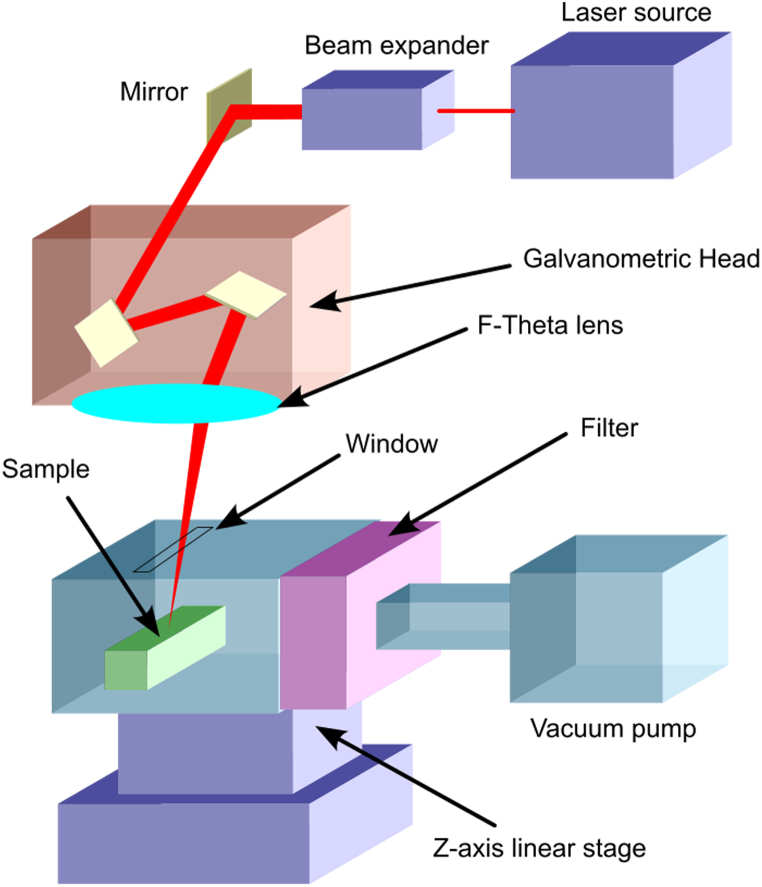
Scheme of the ad hoc receptacle setup to collect samples during laser ablation.

**Fig. 4:**
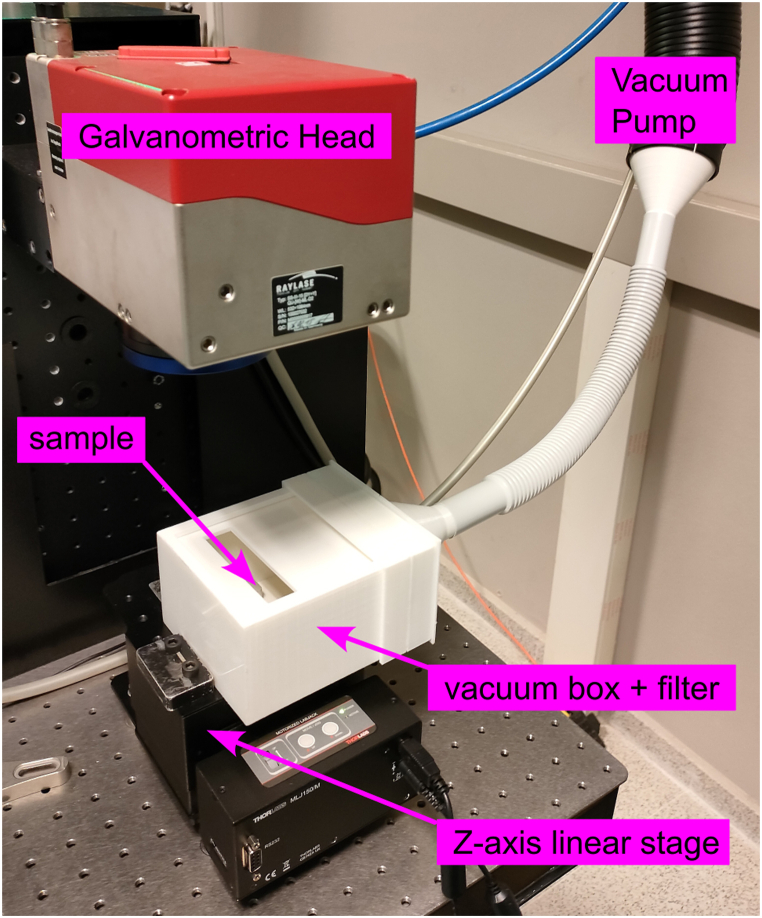
Ad hoc receptacle to collect sample during laser ablation (modified from Pereira et al. (2022)).

The laser utilized in this study was a femtosecond pulsed near-infrared laser, specifically the Spirit system by Spectra-Physics, which operates at an emission wavelength of 1040 nm and a pulse width of less than 400 fs. The intensity profile at the laser output was nearly Gaussian (M2 < 1.2), with a beam diameter of 1.5 mm at the laser head exit and horizontal polarization (greater than 100:1). The pulse rate is adjustable from a single shot to 1 MHz, with a maximum pulse energy of 40 μJ at 100 kHz. The highest mean power output exceeds 4W. A Raylase SuperscanIII-15 two-mirror galvanometric scanner was employed to direct the laser beam in X-Y directions. The beam was focused on the sample surface using an F-theta objective lens with a 160 mm focal length, resulting in a spot size of approximately 30 μm in diameter. To collect dust generated during the laser ablation process, a plastic sample holder was created using 3D printing (Fig. 3, Fig. 4). This custom-designed holder was integrated with the laser laboratory's aspiration system. A mask acting as a filter was placed at the suction tube entrance, causing dust to deposit directly on the outer face of the filter rather than passing through it. In the view of the results we obtained through the present study, we indeed propose this setup as an effective additional protective measure for personnel performing laser ablation on rocks containing NOA. In selecting processing parameters, our objective was to produce sufficient powder for analysis under laboratory conditions, rather than optimizing the laser cleaning technique for removing unwanted layers while preserving the rock's integrity. Consequently, the ablation of serpentinite, metabasite and ophicalcite samples was performed over a 15 mm × 40 mm area, following a pattern of horizontal and vertical lines with a 20 mm separation, at a scan speed of 100 mm/s and maximum laser power (4 W). The ablation pattern was repeated 20 times on the sample, resulting in a total process duration of approximately 2 h (Fig. 3). In this experiment, we used HEPA filters since they are the most widely used filters in various industrial and work environments for air purification, Since the objective of this work was to qualitatively and quantitatively evaluate, in a controlled laboratory environment, the particles emitted during the laser ablation process of samples, it was necessary to design an ad hoc sample holder. This device consists of a box with a window for the laser beam to impinge on the sample surface and an exhaust tube in its upper surface to be connected to the laboratory vacuum system, placed near the laser workstation. A filter was placed before the exhaust tube at a distance of about 10 cm from the sample surface to collect the particles generated during the ablation process. Using this apparatus, samples of approximately 30 x 50 × 10 mm^3^ were laser processed, the ablation plume was confined within the box to avoid dispersion, and the emitted particles were collected in the filter. Laser cleaning is considered a better and more environmentally friendly technique than traditional chemical or mechanical cleaning. Unlike traditional cleaning technologies, it does not use solvents or consumables, is not noisy, and produces less pollution than the former. As a result, laser cleaning can be done safely, especially when the risks are known and the particles released are considered. Filters and exhaust systems prevent harmful particles from being released into the environment, entering the human body or settling on work equipment.

### 2.3 Characterization of retained material

After laser ablation, the HEPA filter retained material powder was characterized by Powder X-Ray diffractometry (PXRD); transmission electron microscopy (TEM) coupled with Energy Dispersive Spectrometry (EDS) and μ-Raman and FTIR spectroscopies. PXRD was performed using a Bruker D8 Advance X-ray diffractometer working at 40 kV and 40 mA. The instrument was equipped with a copper tube and curved graphite monochromator. Scans were recorded in the 2θ range of 3–66°, with a step interval in 2θ of 0.02° and a step-counting time of 3 s/step. The EVA software (DIFFRACplus EVA) was used to identify the mineral phases and experimental peaks were compared with the PDF2 reference patterns. TEM analyses were carried out using a Jeol JEM 1400 Plus (120 kV) equipped with Jeol large-area silicon drift detector SDD-EDS (Jeol, Tokyo, Japan) for microanalyses. EDS spot analyses were conducted on each observed fibre. Analyzed fibrous amphiboles were plotted on the binary diagram Si vs. Mg/(Mg ^+^ Fe^2+^), which are the standard diagrams for the classification of amphiboles [42]. For TEM investigations, the HEPA filter was gently scraped via spatula, and the resulting dust was deposited on a Formvar carbon-coated copper grid. Several TEM micrographs were recorded in order to analyze at least 40 fibres for each sample. Measurements of the fibres were carried out using the OPTIKA PROView image analysis software. In the present work, both regulated asbestos fibres (length >5 μm, width <3 μm, aspect ratio ≥3:1) and non-regulated fibres [1,2] were measured, counted, and classified based on the EDS spectrum and μ-R data. Micro-Raman (μ-R) analyses were carried out using a Jobin Yvon Evolution micro-Raman spectrometer (HORIBA Jobin Yvon, Paris, France), applying the 532 nm laser source. Instrument calibration was carried out utilizing the ∼520.6 cm^−1^ band of a silicon standard. The spectra were acquired by addressing 10 % of the nominal laser power on the bundle of fibres observed directly on a piece of the same filters analyzed under the other techniques, with 1 exposure of 200 s, using the 80 × magnification objective of the microscope. Fourier Transform Infrared Spectroscopy (FTIR) analyses were carried out on powder samples in reflection mode by using a PerkinElmer Spectrum 3 equipped with the PerkinElmer Universal ATR sampling accessory.

## 3 Result

### 3.1 PXRD characterization

Powder X-Ray diffractograms of all collected powders displayed some sharp peaks, indicative of a high level of crystallinity even after laser treatment. Among the non-asbestiform phases, albite was the main detected phase after the ablation. In all retained powder samples, asbestos phases were found in variable amounts (Table 1). Ophicalcite and serpentinite samples (R1, A, A18) provided the highest abundance of serpentine among the detected phases. Sample A19 showed a very low intensity for asbestos. Metabasite samples did not give much information regarding asbestos phases (e.g., tremolite asbestos, actinolite asbestos) probably due to either i) low amount of asbestos (A20), or ii) overload from other non-asbestos phases that appeared in higher amounts. However, when increasing the PXRD intensity some characteristic reflections of asbestos (tremolite asbestos, actinolite asbestos) were detected (Supplementary materials Fig. S1).

**Table 1:** Lithotype and locality of the currently abandoned quarries. Mineralogy of samples before (raw sample) and after laser ablation, as detected by PXRD, TEM, μ-Raman and FTIR. Ab albite, Chl chlorite, Ep epidote, Ms muscovite, Cal calcite, Mag magnetite, Hbl hornblende, f-Atg fibrous antigorite, Tr tremolite asbestos, Act actinolite asbestos, Ctl chrysotile, PS polygonal serpentine, Atg antigorite, Lz lizardite. ∗Regulated asbestos phases.

Sample	Lithotype	Locality	Coordinates Long; Lat	Phases detected before the laser	Phases detected after laser
A19	Metabasite	Conflenti	612563; 4324358	Chl > Ab > Tr∗>Hbl > Cal	Alb > Tr∗>Cal
A20	Metabasite	Conflenti	612477; 4324516	Chl > Ab > Ep > Ms > Act∗>Cal	Chl > Ab > Ms > Act∗
R1	Ophicalcite	Reventino	613598; 4321561	Cal > Chl > Ctl∗>Tr∗	Cal > Ctl∗>Tr∗
A	Serpentinite	Reventino	613598; 4321561	Lz > Ctl∗> PS > Atg > Chl > Mag	Ctl∗>Mag
A18	Serpentinite	Reventino	613598; 4321561	Atg > Lz > PS > Ctl∗> Chl	Ctl∗>f-Atg > Chl

### 3.2 TEM/EDS characterization

**Fig. 5:**
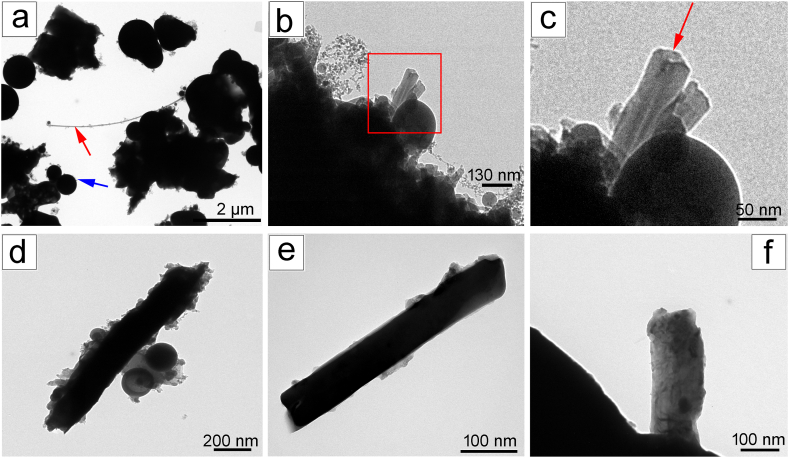
TEM image of asbestos phases after laser treatment: (a) TEM image acquired at low magnification of HEPA filters residues aggregates, amorphous corpuscles (blu arrow) and chrysotile fibre (red arrow), (b) detail of a chrysotile fibre with relative magnified area reported in (c), note the core of chrysotile indicated by red arrow, which runs empty longitudinally along the fibre's axis, (d) fibrous antigorite with spherical amorphous residue around the fibre, (e) actinolite fibre, showing rod-shaped morphology (f) tremolite fibre.

In all samples, TEM image showed mainly the residues of the filter, amorphous corpuscles of spherical morphology (Fig. 5a–d) that we did not characterize to maintain the focus on the asbestos fibre phases. The detected asbestos fibres are tremolite asbestos, actinolite asbestos and chrysotile. Other phases detected in the samples concord with the phases detected by PXRD and can be correlated to the residue of the ablated mother rock (Table 1). At higher magnification ( × 30k magnification), chrysotile and fibrous antigorite could be detected, the individual chrysotile fibres showing the classic tube shape (Fig. 5b). Core of the chrysotile runs along all the length of the fibre (Fig. 5c). The polygonal serpentine fibres (Table 1) consistently exhibited a diameter greater than 100 nm and were wider than chrysotile fibres. Polygonal serpentine displays an overall fibrous habit, its crystal structure consists of polygonal sectors, each featuring a 'lizardite-like' planar TO structure [43,44]. Tremolite asbestos as actinolite asbestos exhibit a prismatic rod-shaped morphology (Fig. 5e and f). Some fibres show fractures transverse to the fibre axis, provoked by the laser, leading to a local decrease in diameter. These fractures were much more evident in amphiboles (tremolite-actinolite) than in chrysotile, probably due to the more flexible nature of chrysotile.

**Fig. 6:**
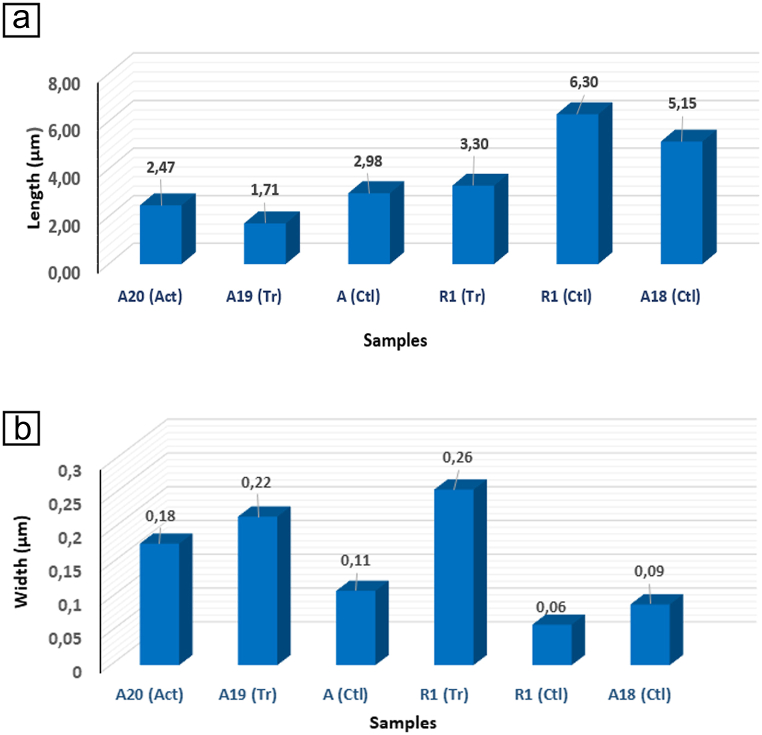
(a) length distribution of samples fibres, as calculated by measuring 40 single fibres for each sample, (b) Width distribution of samples fibres, as calculated by measuring 40 single fibres for each sample. Tr = tremolite, Act = Actinolite, Ctl = chrysotile.

In order to obtain the size distribution of asbestiform fibres detected in the HEPA filter, the length and width of approximately 40 fibres for each sample were measured. Great dimensional variability is observed among the measured fibres (Fig. 6a and b) with a 33.2 % falling within the WHO criteria for respirable fibres. The fibres showed lengths up to a maximum of 6.3 μm (Fig. 6a) with an average value of about 0.98 μm. In terms of width size (Fig. 6b), the fibres had a diameter of less than 0.5 μm, with an average value of fibre width of 0.13 μm. Notably, Wylie and Korchevskiy [45] claimed that asbestos particles with widths less than 0.25 μm should be primarily considered a potent mesothelial carcinogen requiring special attention for public. Overall, the tremolite-actinolite asbestos fibres displayed a wider diameter than the chrysotile ones, while the longest fibres were those of chrysotile. All the detected fibres were chemically characterized through the EDS/TEM spectra and their relative oxide compositions were determined. The detected asbestos fibres are tremolite asbestos, actinolite asbestos and chrysotile (Supplementary materials Table S1). EDS/TEM semiquantitative analysis revealed that chrysotile composition deviates slightly from the ideal formula of Mg end-members, with some percent substitution of Si and Mg by Al and Fe respectively, in agreement with Bloise et al. [29]. The representative chemical formula for chrysotile were (Mg_2.54_Fe_0.19_)(Si_1.92_Al_0.23_)O_5_(OH)_4_ for R1 sample, (Mg_2.54_Fe_0.17_)(Si_1.99_Al_0.15_)O_5_(OH)_4_ for A sample and (Mg_2.59_Fe_0.27_)(Si_1.91_Al_0.19_)O_5_(OH)_4_ for A18 sample (Supplementary materials Table S1). Moreover, EDS/TEM analyses allowed to perform the discrimination between tremolite asbestos and actinolite asbestos (Supplementary materials Table S1), for this purpose, the average compositions of the analyzed fibrous amphiboles were plotted on the binary diagram Si vs. Mg/(Mg ^+^ Fe^2+^), which are the standard diagrams for the classification of amphiboles [42]. The representative chemical formula of tremolite asbestos written on the basis of 23 oxygen atoms were: Ca_1.88_Na_0.04_(Mg_4.84_Fe_0.34_)[(Si_7.86_Al_0.02_)O_22_]OH_2_ for A19 sample, and Ca_2.31_(Mg_4.58_Fe_0.21_)[(Si_7.73_Al_0.17_)O_22_]OH_2_ for R1 sample and actinolite asbestos Ca_2.12_(Mg_4.46_Fe_0.56_)[Si_7.88_O_22_]OH_2_ for A20 sample. It is noteworthy that the chemical composition of the fibres does not vary by laser ablation [6,29,32,41].

### 3.3 μ-Raman

**Fig. 7:**
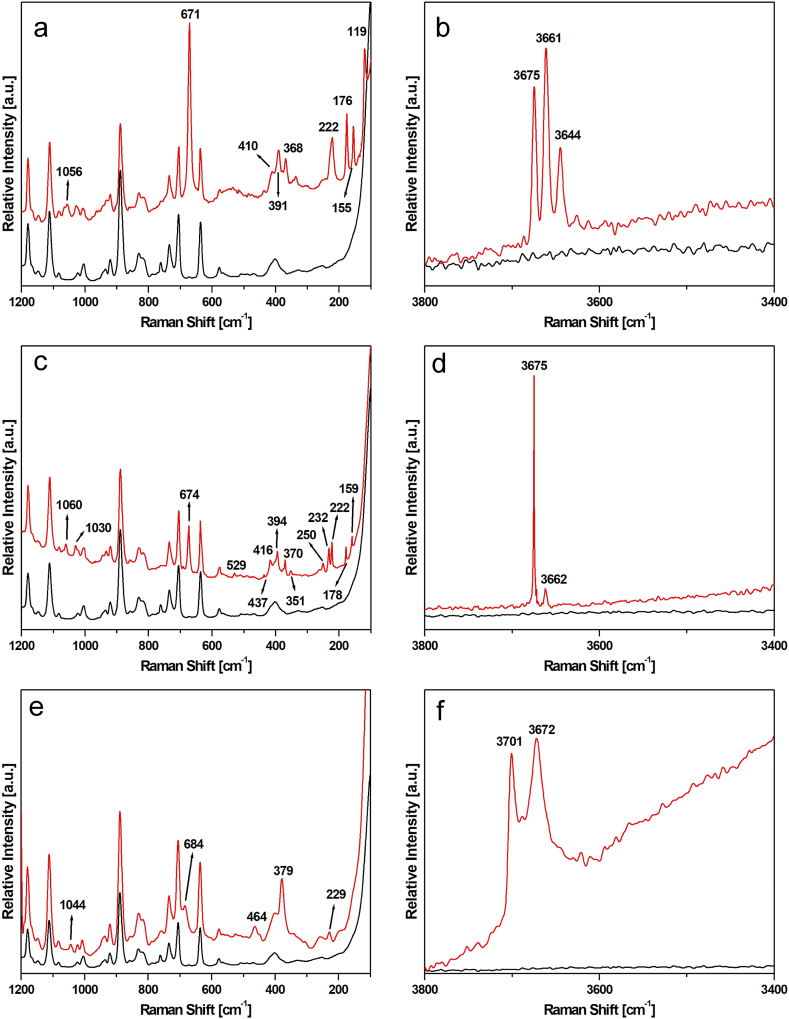
(a) Raman spectrum recorded in the 1200-100 cm^−1^ spectral range on a bundle of fibres ascribed to actinolite (sample A20), (b) high wavenumber spectral range related to the spectrum of actinolite is reported in Fig. 7a, (c) Raman spectrum was recorded in the 1200-100 cm^−1^ spectral range on a bundle of fibres ascribed to the tremolite mineral phase (Sample R1), (d) high wavenumber spectral range related to the spectrum of tremolite is reported in Fig. 7c, (e) Raman spectrum was recorded in the 1200-100 cm^−^^1^ spectral range on a bundle of fibres ascribed to the serpentine phase antigorite (Sample A18). (f) High wavenumber spectral range related to the spectrum of antigorite is reported in Fig. 7d. Black line, reference spectrum of HEPA filter.

**Fig. 8:**
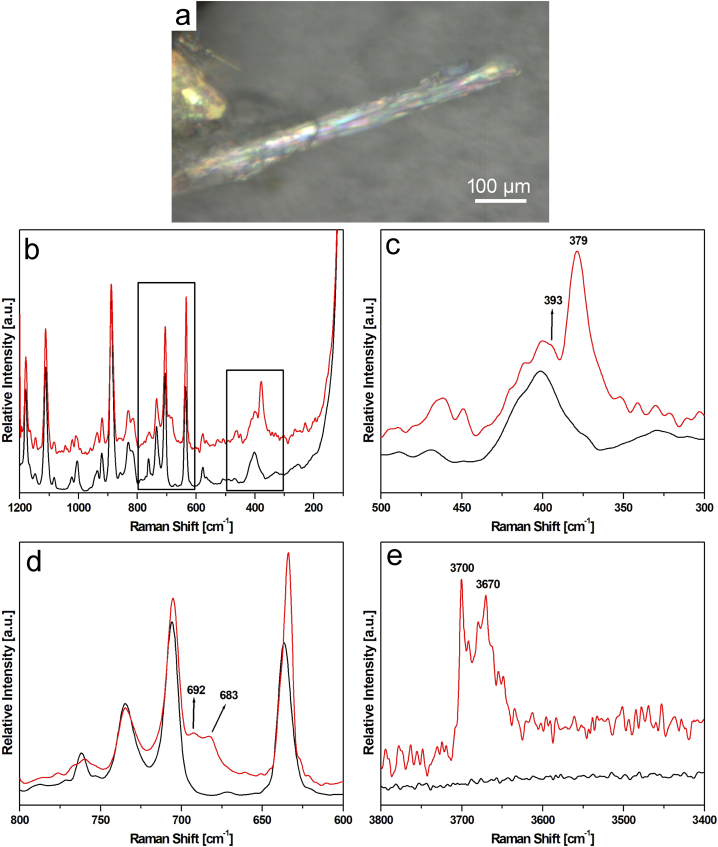
(a) Bundle of fibres observed on the filter (Sample A18), (b) Raman spectrum obtained on the bundle of fibres of Fig. 8a in the 1200-100 cm^−1^ spectral range; the black boxes indicate the spectral ranges reported in Fig. 8c and d, (c) particular of the 500-300 cm^−1^ spectral range of the Raman spectrum is reported in Fig. 8b, (d) particular of the 800-600 cm^−1^ spectral range of the Raman spectrum is reported in Fig. 8b, (e) high wavenumber spectral range reported in Fig. 8b; in this case, the indicated Raman bands allow to identify the mineral phase as chrysotile; black line, reference spectrum of HEPA filter.

In Fig. 7a–f representative Raman spectra obtained from the analyzed fibres are reported. Note that in all graphs, the spectrum of the HEPA filter substrate (black line) has been systematically added to better distinguish the bands exclusively deriving from the mineral phases. In all spectra, two peaks lying near the Si-O_b_-Si symmetric stretching of the asbestos phases are evident: these bands are located at 706 and 636 cm^−1^ [46]. From these signals, it is therefore possible to recognize the signature deriving from the bundles of fibres. Moreover, to better determine the mineral phase, the range of higher wavenumbers (3800-3400 cm^−1^) was considered (Fig. 7b–d and f). Different spectral features were observed. In the first two cases, a band lying between 670 and 675 cm^−1^ is detected: this signal, coupled with the ones ascribed to the Si-O_b_-Si antisymmetric scattering (in the Raman range 1000–1100 cm^−1^) allows to surely identify with high precision the amphibolic asbestos phases. In the case of the spectrum reported in Fig. 7a, the first band is located at 671 cm^−1^, whereas the second one lies at 1056 cm^−1^, so the mineral phase associated with this elongated phase was actinolite. In Fig. 7c, the other feature relative to the Ca-amphibolic phase is reported: in this case, the bands are at 674, 1060, and 1030 cm^−1^, characteristic of tremolite asbestos [47,48]. Because the low wavenumbers range shows different signals relative to the HEPA substrate, high wavenumbers range was also acquired, to definitively identify the mineral phase. As expected, in the first case, three Raman bands at 3675, 3661, and 3644 cm^−1^ were detected (Fig. 7b), whereas in the second case only one very strong signal at 3675 cm^−1^ and a very weak peak at 3662 cm^−1^ were observed (Fig. 7d). This Raman range gives useful information about the OH bonds in the amphibole structure. In the tremolite-actinolite series these signals are ascribed to the linkage of the elements that occupy octahedral sites (Mg and Fe) with OH ions [49,50]. In particular, the band at 3675 cm^−1^ is relative to the stretching of the OH groups linked to MgMgMg triads, the one at about 3660 cm^−1^ is ascribed to the ones bonded to Fe^2+^MgMg triads and lastly, the signal located at about 3645 cm^−1^ is relative to the hydroxides attached to Fe^2+^Fe^2+^Mg ions [50]. So, considering both low and high wavenumbers Raman ranges, it is possible to undoubtedly ascribe the signals to the presence of actinolite or tremolite asbestos directly on the filters. In Fig. 7e and f, a third kind of spectral feature is reported. In this case, in the low wavenumber range a minor number of Raman bands are detected: the positions of these bands allow to define a serpentine phase and, considering the Raman bands ascribed to the symmetric and antisymmetric stretching of Si-O_b_-Si bonds (at about 680–690 cm^−1^ and 1040-1110 cm^−1^ respectively), to identify the mineral phase as antigorite [51,52]. This conclusion is confirmed also considering the positions of the bands related to the OH bonds [53,54]: in fact, the band at 3701 and 3672 cm^−1^ are ascribed to the antigorite phase. Decreasing the time of exposure from 200 s to 30 s, it is possible to detect some little differences in the acquired bands (Fig. 8a and b). In fact, on bundles of fibres like the one reported in figure Fig. 8 a, it is possible to observe some weak signals lying at 393 cm^−1^ (Figs. 8c) and 693 cm^−1^ (Fig. 8d). These signals are consistent with the Raman spectrum of chrysotile, another serpentine phase [48,52,53]. Moreover, in the high wavenumbers spectral range (Fig. 8e), it is possible to observe a different intensity ratio between the bands lying at 3701 and 3672 cm^−1^: this behavior may be ascribed to the presence of chrysotile. The more prominent bands of antigorite mineral phase might be explained by two hypotheses: (i) analyzing many particles, antigorite is present in higher percentages compared to chrysotile, (ii) antigorite gives more intense Raman spectra than chrysotile working in the same operational condition. It is important to note that only chrysotile is legally defined as an 'asbestos' phase [2], while fibrous antigorite is not classified as asbestos. Therefore, accurately identifying and distinguishing fibres as either fibrous antigorite or chrysotile is crucial from both legal and health risk perspectives.

### 3.4 FTIR characterization

**Fig. 9:**
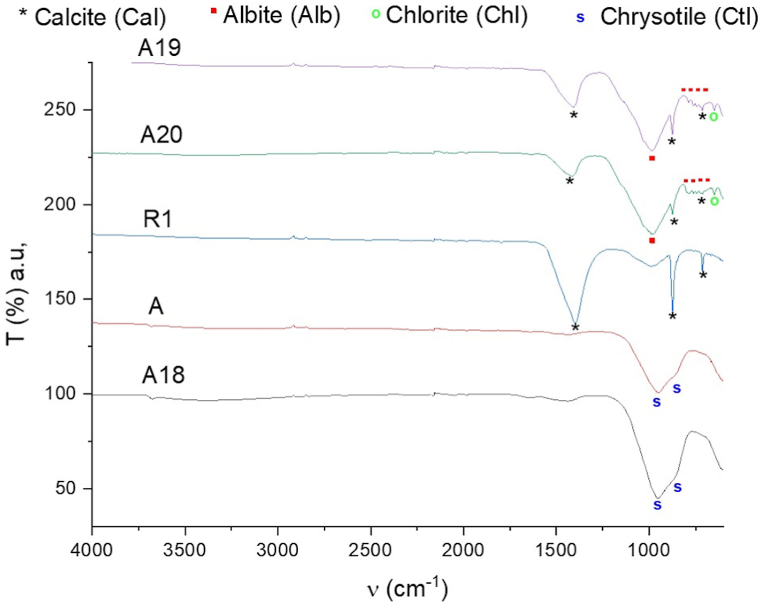
FTIR-ATR spectra of all samples with attempted assignments.

To complete the characterization of the collected powders from the inserted filters, FTIR-ATR spectra were registered on all samples. Although less accurate than μ-Raman, FTIR-ATR allows the assignment of certain bands to specific mineral phases and has the tremendous advantage of being immediate. All spectra are reported in Fig. 9 and the obtained results are consistent with both PXRD and μ-Raman characterization. Worthy of note is the fact that in all observed powders, signals relative to the HEPA filter constituents have not been observed. The reason for this is that, on the broader macroscale of FTIR-ATR with respect to the μ-Raman technique, the quantity of filter residue is too low to be observed in comparison to the inorganic mineral content. Starting from the spectrum registered for sample R1, three characteristic bands are observed. The first two bands are straight and centered respectively at 713 and 872 cm^−1^ while the third and wider band is located at 1396 cm^−1^. These signals belong to the characteristic FT-IR signature of the Calcite (Cal) phase [55]. On the R1 spectrum, another wider band of lower intensity is observed at ca. 990 cm^−1^, probably relative to the Si-O-Si framework vibrations of an unidentified silicate phase of lower content. The three characteristic bands of calcite are also observed in samples A19 and A20 which present nearly superimposable features. Indeed, for these two samples, the calcite signals are lower in intensity compared to the wide and intense band of the Si-O-Si framework centered at 990 cm^−1^, which is accompanied by a set of four consecutive bands (marked with red dots in Fig. 9) at 720, 740, 760 and 790 cm^−1^ that are specific to the signals observed for the albite (Alb) phase [56]. According to PXRD analysis, both samples A19 and A20 should also contain chlorite which might be observed in the FTIR-ATR spectra as a weak signal at 645 cm^−1^ whose attribution cannot be however confirmed by other significative bands (Reference spectrum of Chlorite is obtainable from the Department of Geology at University of Tartu web site). Finally, samples A and A18 present identical spectral features characterized by two consecutive wider bands centered at 925 and 990 cm^−1^ which could be reconducted to the presence of chrysotile which should display three consecutive characteristics bands at 921, 1010 and 1100 cm^−1^ according to Jovanovski and Makreski [57]. In samples R1 and A19, the tremolite phase has not been observed through FTIR-ATR spectroscopy, but this could be easily attributed to the fact that characteristics signals of tremolite [58] might be covered by the higher content of the other mineral phases.

## 4 Discussion

The fibrous materials retained in HEPA filters after laser treatment were characterized and confirmed that such process, as probably any operational process of NOA-containing rocks, may cause the dispersion of asbestos fibres (chrysotile, tremolite, antigorite) into the air. Today, the attention on air contamination by asbestos from different sources is claiming increasing information. Indeed, the polluted air may pose a risk related to the air migration of fibres. Asbestos is well-known for triggering serious health risks that can lead to several debilitating lung diseases, including asbestosis lung cancer and mesothelioma [4]. The World Health Organization (WHO) estimates that approximately 107,000 people worldwide die each year from asbestos-related diseases [1], including around 43,000 from mesothelioma [59]. The identification of asbestosis as a disease associated with the inhalation of airborne asbestos fibres gradually emerged during the 20th century as a result of medical research and epidemiological studies. Asbestos fibres may enter the body through inhalation or oral intake. It has been demonstrated that inhalation exposure produces the most damaging outcomes [60]. Inhaled asbestos fibres can become trapped into the lungs and surrounding tissues. Over time, the immune system forms scar tissue around these fibres, leading to the development of 'asbestos bodies', which can obstruct lung function and result in symptoms like breathing problems, persistent coughing, and even respiratory failure [61]. Exposure to asbestos can occur in two forms: occupational exposure, whereby workers encounter asbestos during working activities and environmental exposure, caused by the natural erosion of asbestos deposits or rocks containing asbestos as its major component. In addition to occupational and environmental exposure, para-occupational exposure refers to accidental exposure to hazardous materials like asbestos for individuals not directly involved in such work. For example, asbestos fibres may be brought home on clothing or work tools of family members. Although mesothelioma cases were reported, allegedly caused by such exposures, the literature lacks quantitative data [62]. Despite being banned in 55 countries worldwide, asbestos continues to pose a real health risk in countries such as Russia and China, where asbestos is widely produced and handled [63]. As demonstrated in this work, every time a rock containing asbestos is disturbed through a cut that can be made with diamond saws or lasers, a significant quantity of asbestos fibres is released into the air. Furthermore, it has been herein demonstrated that both the chemical composition and the dimensions of the fibres are not altered after laser treatment. Morpho-dimensional analysis of tremolite asbestos, actinolite asbestos and chrysotile showed variability in fibre dimensions, with a 33.2 % falling within the WHO criteria for respirable fibres. Consideration must also be given to the carrier function that air can have for fibres, which can cause their transport, and possible accumulation, in areas even distant from the original source of contamination [64]. From these findings, it is clear the importance of assessing asbestos pollution in the air where rocks containing asbestos are disturbed (cut, worked, cleaned) or left to stand outdoors as in abandoned quarries or working sites and how it can potentially be a risk to humans. From a mineralogical point of view, tremolite asbestos represents the most abundant asbestos phase in the studied case, followed by chrysotile and actinolite asbestos. Despite these observations, it is essential to investigate the topic further. Asbestos fibres can be released into the air through manipulation of asbestos or working with asbestos-containing material, which can represent a secondary source of airborne fibres. Micro-Raman spectroscopy [[65], [66], [67]] and FTIR-ATR [68,69] are simple and quick methods to precisely characterize the mineral phase associated to the different elongated morphologies. In fact, these techniques do not need specific preparation of the samples. While FTIR-ATR can give a prompt first analysis with specific signatures of the mineral phases, we observed that characteristic bands could be hidden underneath signals coming from other mineral phases. Instead, μ-Raman can identify the single bundles of fibres and by focusing the laser beam to target them for the analysis, thanks to the microscope annexed to the spectroscope, it is possible to ascertain the presence of the harmful fibres. Indeed, the obtained spectra are a “fingerprint” of the different phases, allowing their precise identification. In this work, it has been shown that it is possible to easily discriminate between actinolite asbestos and tremolite asbestos also directly on HEPA filters routinely used for asbestos environmental samplings applying μ-Raman spectroscopy. For this specific study, particularly useful is the Raman range where the OH bands lie. Moreover, considering both the low and the high wavenumber ranges, the technique revealed itself very useful in the discrimination of serpentine phases, in the present work between chrysotile and fibrous antigorite. This last aspect is very important in the field of environmental studies, because both the phases crystalize in elongated morphologies, but only chrysotile is defined by Law as an asbestos phase. The technique is very useful in serpentine phase discrimination also in case of their contemporary presence inside the same bundles of fibres, as reported in this work.

## 5 Conclusions

The combination of μ-Raman and FTIR leads to a precise diagnosis of the nature of asbestos fibres contained in a non-environmental matrix (i.e., HEPA filters). The TEM study allows us to investigate the asbestiform (or non-asbestiform) morphology. The use of this combined system allows you to carry out an accurate diagnosis of the type of material treated by the HEPA filters. HEPA filters contained breathable (regulated) fibres (33 %) according to the WHO (1997) counting criteria (length ≥5 μm, width ≤3 μm, length/width ratio ≥3:1) [70].

The analyses conducted in this study revealed that asbestos fibres present in HEPA filters after laser ablation exhibit chemical and morpho-dimensional characteristics that define their pathogenicity. Among the minerals examined within the asbestos group, three out of the six minerals (tremolite asbestos, actinolite asbestos, chrysotile) were identified. The release of asbestos during laser ablation processes, such as cleaning and texturizing, poses a significant threat to occupational safety. HEPA filters play a crucial role in capturing various types of particles released during laser ablation, that may be employed in different processes (cleaning, cutting, drilling …) which involve rocks containing asbestos, such as serpentinites, metabasiti and ophicalcite. The present study reveals also that incorporation HEPA filters as a cost-effective preventive measure and integrating a filtration system into the ablation equipment, along with workers using masks, can effectively reduce the risk of fibre inhalation during cleaning operations aimed at preserving and safeguarding monuments and historical structures. Sharing these findings with workers through outreach initiatives can trigger a self-preservation instinct, safeguarding their health from exposure to these hazardous materials. Furthermore, further comprehensive research on samples containing a higher concentration of fibrous minerals is imperative. Providing a detailed explanation of how these fibres can be fatal without proper preventive measures in place can ultimately save lives.w

## CRediT authorship contribution statement

**Andrea Bloise:** Writing – review & editing, Writing – original draft, Visualization, Software, Resources, Investigation, Funding acquisition, Formal analysis, Data curation, Conceptualization. **Francesco Parisi:** Software, Formal analysis, Data curation. **Mauro Francesco La Russa:** Writing – review & editing, Validation, Formal analysis, Data curation. **Carmine Apollaro:** Writing – review & editing, Supervision, Methodology, Data curation. **Nicolas Godbert:** Writing – review & editing, Writing – original draft, Supervision, Software, Formal analysis, Data curation. **Iolinda Aiello:** Visualization, Software, Methodology, Data curation. **Eugenia Giorno:** Software, Methodology, Formal analysis, Data curation. **Alessandro Croce:** Supervision, Methodology, Formal analysis, Data curation. **Laura Cagna:** Methodology, Formal analysis, Data curation. **Ana Jesús López:** Supervision, Software, Formal analysis, Data curation. **Alberto Ramil:** Validation, Software, Formal analysis, Data curation. **Dolores Pereira:** Writing – review & editing, Validation, Funding acquisition, Formal analysis, Data curation.

## Compliance with ethical standards

Ethical Human Participants and/or Animals Not applicable, no human participants and/or animals were used in this research.

## Funding

This research was funded by 10.13039/100026280BRIC 2022 project; CUP: B87G23000090005 and by PID2021-123948OB-100, funded by 10.13039/501100004837MCIN/10.13039/501100011033AEI/10.13039/501100011033 and by the 10.13039/501100008530ERDF "A way of making Europe" of the 10.13039/501100000780European Union.

## Declaration of competing interest

The authors declare that they have no known competing financial interests or personal relationships that could have appeared to influence the work reported in this paper.
