# Supplementary material for: Evaluation of asbestos dispersion during laser ablation of rocks containing Naturally Occurring Asbestos (NOA)
Source: Heliyon. 2024 Oct 22;10(21):e39624. doi: 10.1016/j.heliyon.2024.e39624 (PMC11546477; doi:10.1016/j.heliyon.2024.e39624)
Supplement: Multimedia component 1 [file mmc1.docx]

**Supplementary Materials**

**Evaluation of asbestos dispersion during laser ablation of rocks containing Naturally Occurring Asbestos (NOA)**

Andrea Bloise^a,b,*^, Francesco Parisi^a^, Mauro Francesco La Russa^a^, Carmine Apollaro^a^, Nicolas Godbert^c,d^, Iolinda Aiello^c,d^, Eugenia Giorno^c,d^, Alessandro Croce^e,f^, Laura Cagna^e^, Ana Jesús López^g^, Alberto Ramil^g^, Dolores Pereira^h^

^a^Department of Biology, Ecology and Earth Sciences, University of Calabria, 87036, Rende (CS), Italy

^b^University Museum System – SiMU, Mineralogy and Petrography Section, University of Calabria, 87036, Rende (CS), Italy

^c^MAT_InLAB (Laboratory of Inorganic Molecular Materials), Center of Excellence CEMIF.CAL,LASCAMM CR-INSTM of Calabria, Department of Chemistry and Chemical Technologies, University of Calabria, 87036, Rende (CS), Italy

^d^LPM-Materials Preparation Laboratory, STAR-Lab, University of Calabria, Via Tito Flavio, 87036 Rende (CS), Italy

^e^Department of Science and Technological Innovation, University of Eastern Piedmont, Viale Teresa Michel 11, 15121 Alessandria, Italy

^f^ Research Laboratories, Research, Training and Innovation Infrastructure, Department of Integrated Research and Innovation Activities (DAIRI), SS. Antonio e Biagio e Cesare Arrigo University Hospital, Via Venezia 16, 15121 Alessandria, Italy

^g^Ferrol Industrial Campus, Universidade da Coruña, 15471 Ferrol, Spain

^h^Department of Geology, University of Salamanca, 37008 Salamanca, Spain

^⁎^Corresponding author: andrea.bloise@unical.it

According to the dimensional definition of the [World Health Organization (1997)](javascript:;) and the literature (e.g., [Belluso et al., 2017](javascript:;); [NIOSH 2011](javascript:;)), in this paper we use the definitions listed below:

- Fiber: inorganic particle with length ≥ 5 μm, width ≤ 3 μm, length/width (aspect ratio) ≥ 3:1, parallel sides when seen in two dimensions, perpendicularly to fiber axis.
- Fibril: a single mineral fiber that cannot be further separated longitudinally into smaller components (without losing the fibrous properties or appearances).
- Any mineral particle with a minimum aspect ratio of 3:1 is considered an elongated mineral particles (EMP)
- Asbestiform: adjective for fibrous non-asbestos classified having the “fiber” dimensions and at least one of the asbestos properties, such as flexibility, splitting, and so on.
- Fiber bundle: parallel aggregate of mineral fibers.

**Fig. S1**. XRPD of raw samples A, A18, A19, A20, R1 (black line) and after laser ablation (blue line).

**Table S1.** EDS/TEM datasheets. Average as calculated on 4 single spot analysis.

|  | Tremolite A19 | Actinolite A20 | Tremolite R1 | Chrysotile R1 | Chrysotile A | Chrysotile A18 |
| --- | --- | --- | --- | --- | --- | --- |
| Oxide |  |  |  |  |  |  |
| MgO | 24.4 | 22.1 | 22.8 | 42.1 | 42.4 | 42.9 |
| SiO_2_ | 59.0 | 58.2 | 57.3 | 47.5 | 49.9 | 47.2 |
| CaO | 13.2 | 14.7 | 15.9 | 0 | 0 | 0 |
| FeO | 3.1 | 5 | 2.8 | 5.5 | 4.9 | 5.9 |
| Na_2_O | 0.1 | 0 | 0 | 0 | 0 | 0 |
| Al_2_O_3_ | 0.1 | 0 | 1.0 | 4.8 | 2.8 | 3.9 |
| Cations calculated on the basis of 23 oxygen atoms | | | | Cations calculated on the basis of 7 oxygen atoms | | |
| Mg | 4.84 | 4.46 | 4.58 | 2.54 | 2.54 | 2.59 |
| Si | 7.86 | 7.88 | 7.73 | 1.92 | 1.99 | 1.91 |
| Fe | 0.34 | 0.56 | 0.21 | 0.19 | 0.17 | 0.27 |
| Na | 0.04 | 0 | 0 | 0 | 0 | 0 |
| Al | 0.02 | 0 | 0.17 | 0.23 | 0.15 | 0.19 |
| Ca | 1.88 | 2.12 | 2.31 | 0 | 0 | 0 |
